# Supplementary material for: Achieving High Activity and Long-Term Stability towards Oxygen Evolution in Acid by Phase Coupling between CeO2-Ir
Source: Materials (Basel). 2023 Nov 1;16(21):7000. doi: 10.3390/ma16217000 (PMC10650327; doi:10.3390/ma16217000)
Supplement: Supplementary file 1 [file materials-16-07000-s001.zip › materials-2656031-supplementary.pdf]

Support Information

Achieving High Activity and Long-Term Stability towards Oxygen Evolution in Acid by Phases Coupling between CeO_2-Ir

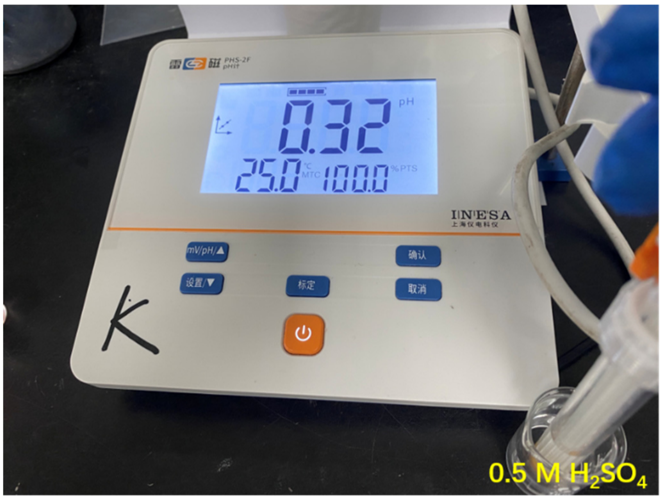

Figure S1. The pH of 0.5 M H_2SO_4 at room temperature.

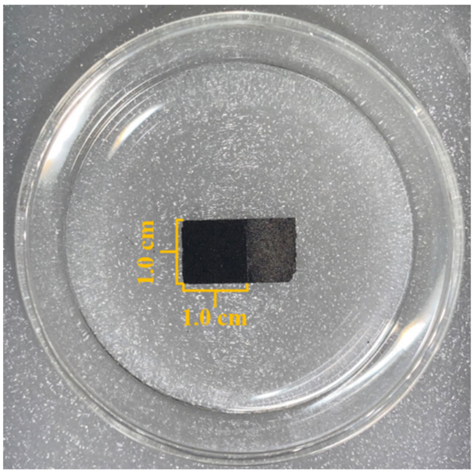

Figure S2. Photograph of catalyst-loaded carbon paper.

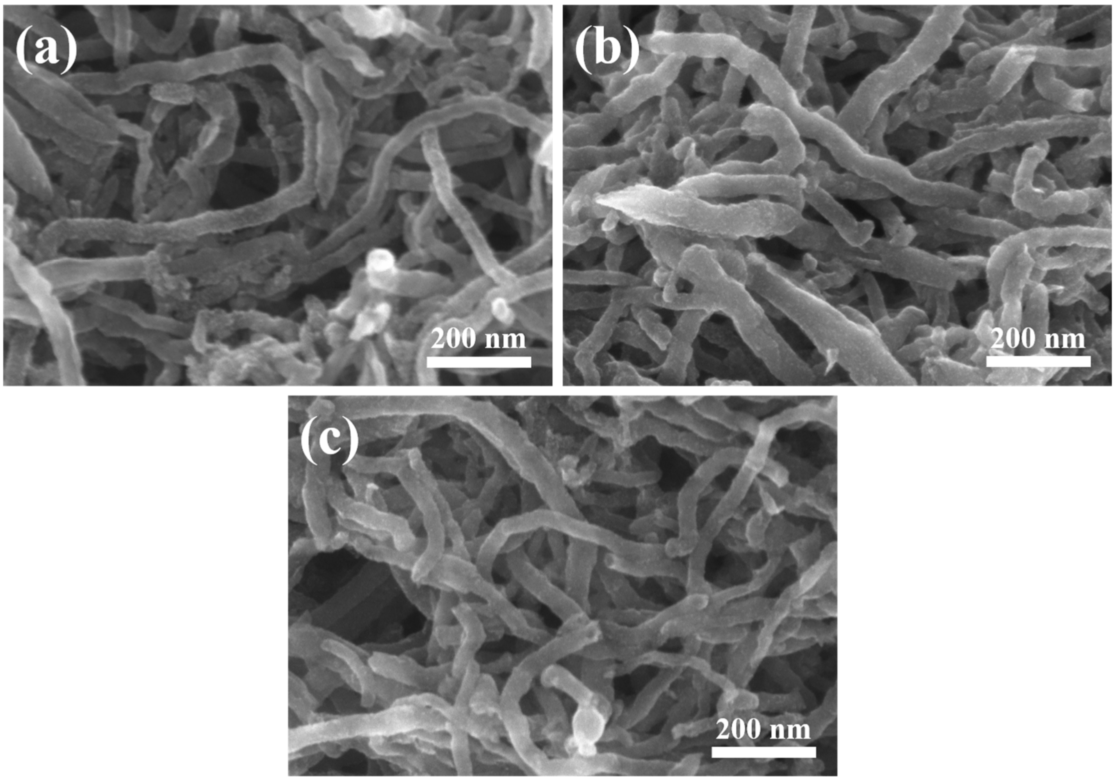

Figure S3. SEM images of (a) CeO_2-Ir/CNTs, (b) Ir/CNTs and (c) CeO_2/CNTs.

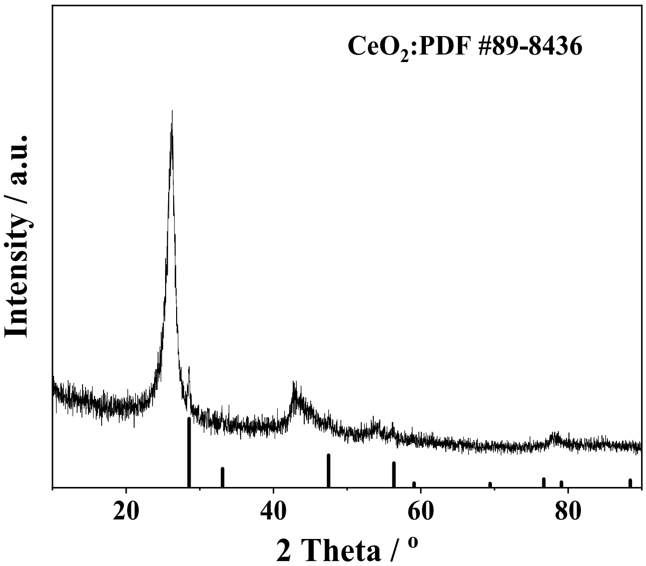

Figure S4. XRD pattern of CeO_2/CNTs.

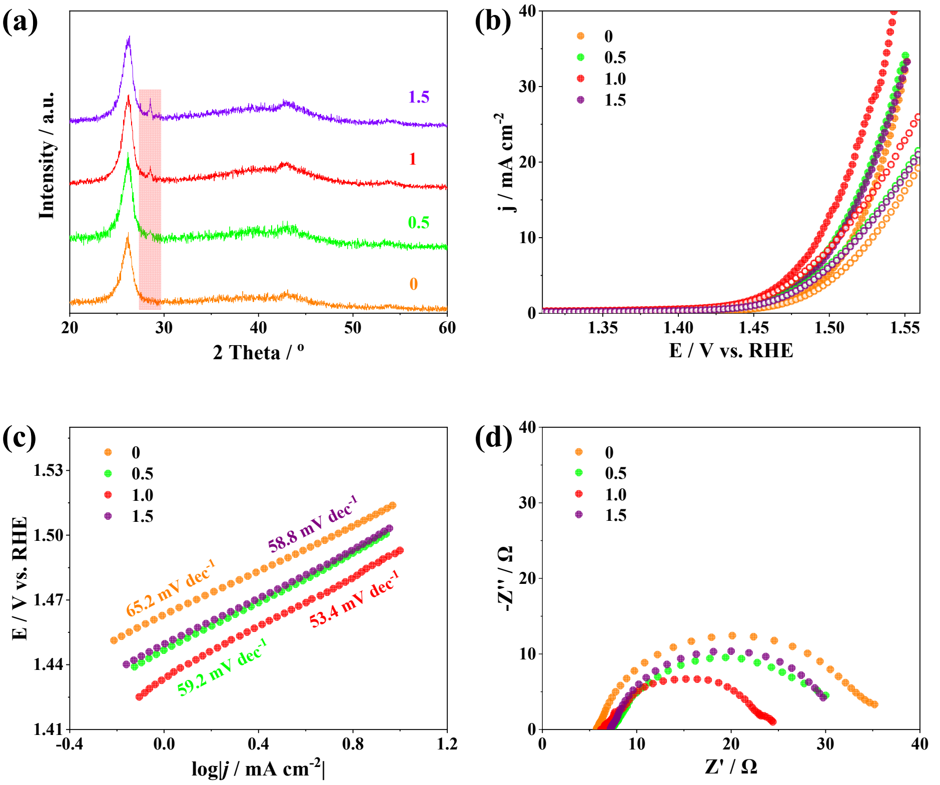

Figure S5. (a) XRD patterns, (b) LSVs (solid-dot line: with iR compensation), (c) Tafel plots and (d) EIS Nyquist plots of xCeO_2-Ir/CNTs with different CeO_2 content.

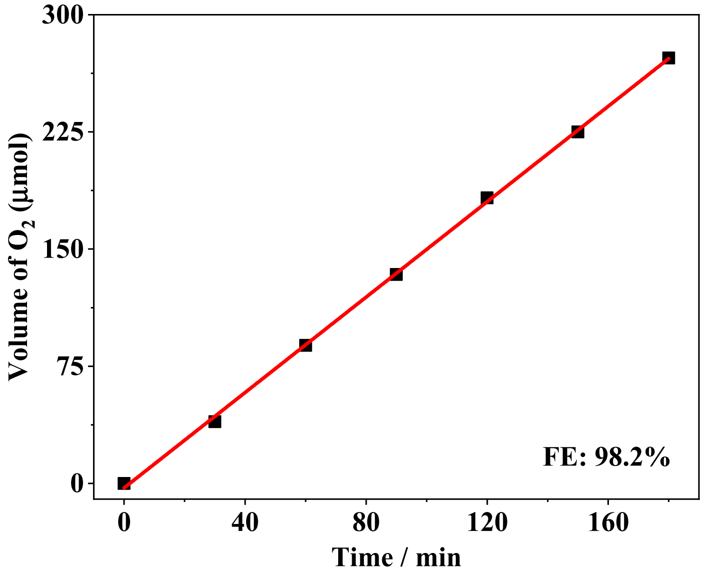

Figure S6. The time-dependent volume of O_2 towards OER using CeO_2-Ir/CNTs as anode. The red line represents a fitted line, while square data points denote the volume of O_2 generated at 30-minute intervals.

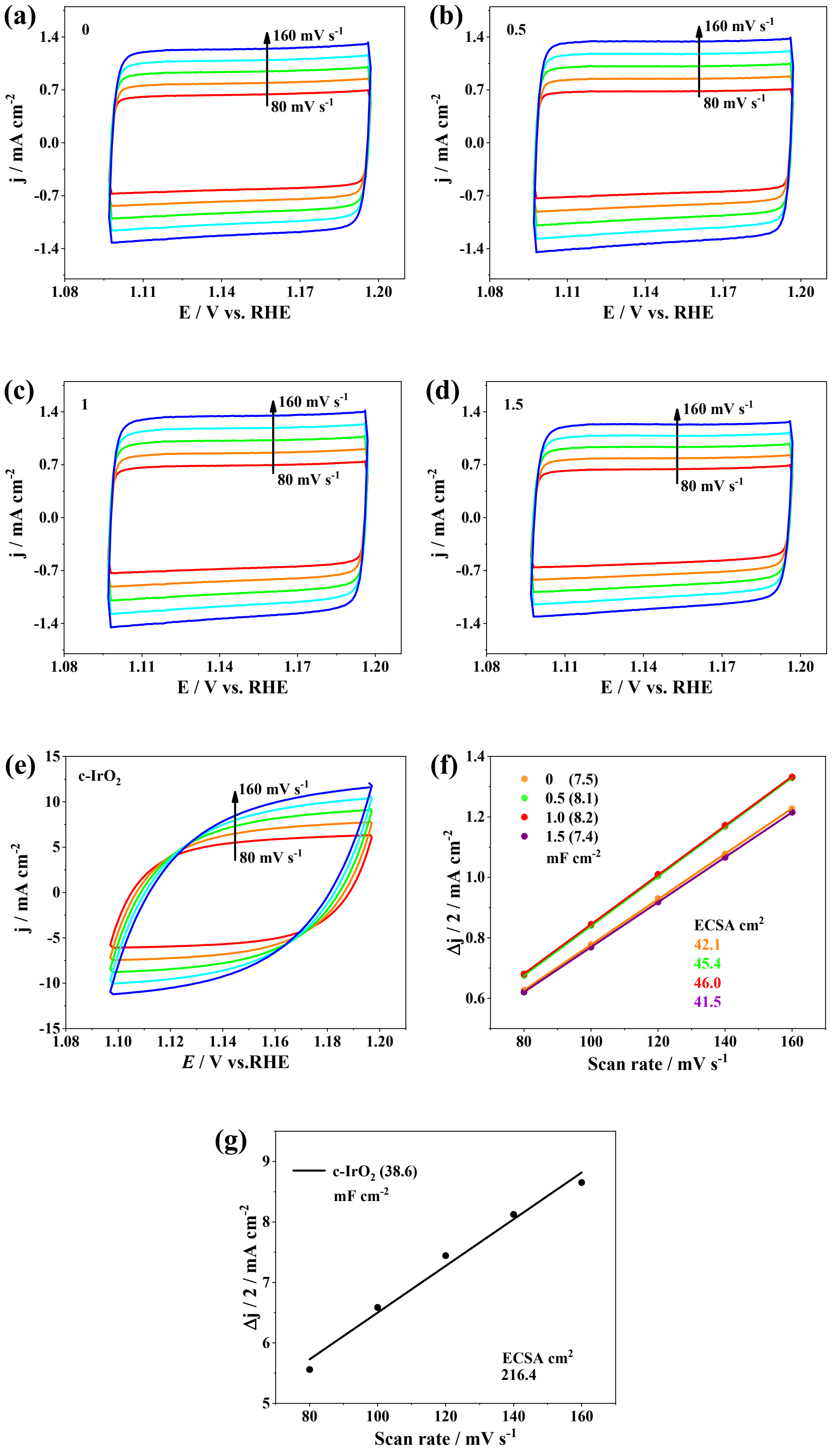

Figure S7. CVs recorded at different scan rates in the region of 1.1-1.2 V of (a-d) $xCeO_2$-Ir/CNTs with different CeO_2 content and (e) c-IrO_2. (f) and (g) C_{dl} plots and ECSAs of the catalysts.

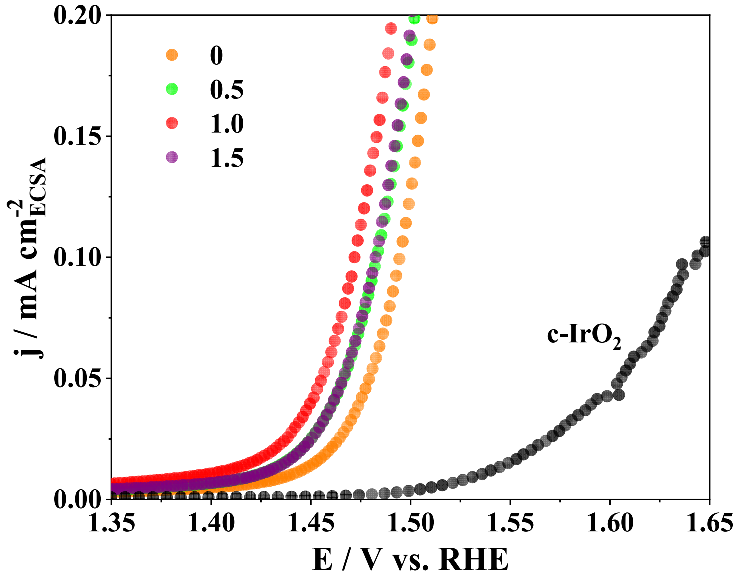

Figure S8. ECSA-normalized LSVs of xCeO_2-Ir/CNTs and c-IrO_2.

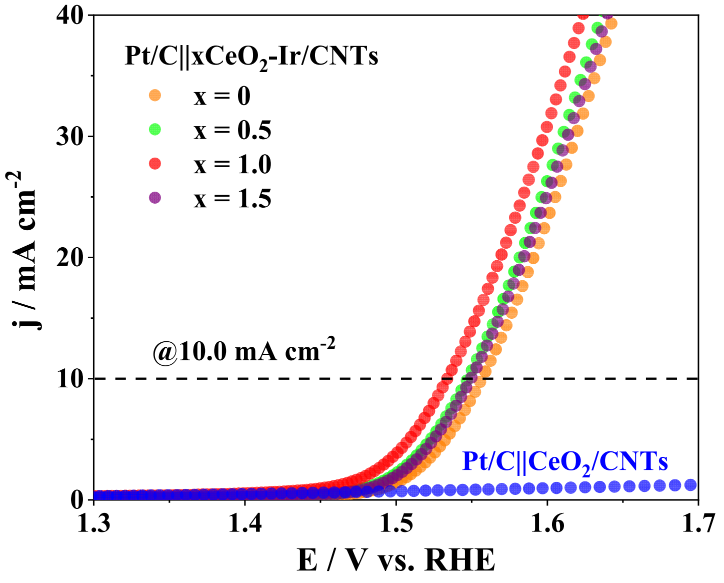

Figure S9. LSVs of Pt/C||xCeO_2-Ir/CNTs and Pt/C||CeO_2/CNTs.

Table S1. Composition analysis of $xCeO_2$-Ir/CNTs.

$xCeO_2$-Ir/CNTs	Mass Concentration / mg L^{-1}		Atomic Concentration / mmol L^{-1}		Atomic ratio
	Ce	Ir	Ce	Ir	Ce:Ir
x = 0	0	56.161	0	0.292	0
x = 0.5	20.635	56.772	0.147	0.295	0.498
x = 1	41.327	56.568	0.295	0.294	1.003
x = 1.5	62.006	56.572	0.443	0.294	1.507

Table S2. Analysis of EDS spectrum.

Element	Atomic Fraction (%)	Mass Fraction (%)
C	94.70	84.94
O	4.52	5.41
Ce	0.39	4.07
Ir	0.39	5.58

Table S3. OER performance comparison of the CeO_2-Ir/CNTs with the catalysts reported in acid media.

Catalyst	Electrolyte	Mass loading	η_{10} (mV)	Stability (h @η_{10})	Mass activity (A g_{Ir}^{-1}@V)	Ref.
CeO_2-Ir/CNTs	0.5 M H_2SO_4	~0.20 mg cm^{-2}	262.9	60	2542.3 @1.53	This work
Li-IrO_x	0.5 M H_2SO_4	0.125 mg cm^{-2}	290	10	100 @1.52	[1]
IrO_2@Ir/TiN	0.5 M H_2SO_4	0.379 mg·cm^{-2}	265	6	480.4 @1.6	[2]
Ir@N-G-750	0.5 M H_2SO_4	23 μg·cm^{-2}	303	20*	2420 @1.6	[3]
IrGa/N-rGO	0.5 M H_2SO_4	-	275	-	-	[4]
Sr_2IrO_4	0.5 M H_2SO_4	0.404 mg cm^{-2}	263			[5]
IrO_2/V_2O_5	0.5 M H_2SO_4	0.1 mg_{Ir} cm^{-2}	266	20	287 @1.53	[6]
IrCo NRAs	0.5 M H_2SO_4	-	296.9	15	315.5 @1.51	[7]
Ru@IrO_x	0.5 M H_2SO_4	~0.051 mg_{oxide} cm^{-2}	282	24	644.8 @1.56	[8]
Ir-CeO_2-C	0.5 M H_2SO_4	1 mg cm^{-2}	283	60	21.95 @1.53	[9]
$IrO_x/SrIrO_3$	0.5 M H_2SO_4	-	~270	30	-	[10]
$IrMoO_x$	0.5 M H_2SO_4	0.816 mg_{oxide} cm^{-2}	267	30	-	[11]
$IrNiO_x$	0.5 M H_2SO_4	10.2 μg_{Ir} cm^{-2}	~309	-	170 @1.50	[12]
Ir_6Ag_9 NTs/C	0.5 M H_2SO_4	13.3 ug_{Ir} cm^{-2}	285	6	-	[13]
La_2LiIrO_6	0.5 M H_2SO_4	0.255 mg_{oxide} cm^{-2}	278	-	-	[14]

*denotes the OER durability tested in 0.1 M $HClO_4$.

Table S4. Component analysis of CeO_2-Ir/CNTs before and after OER.

CeO_2-Ir/CNTs				
	Before OER		Post OER	
Element	Ce	Ir	Ce	Ir
Mass concentration / mg L^{-1}	41.327	56.568	29.479	54.742
Atomic Concentration / mmol L^{-1}	0.295	0.294	0.210	0.285
Atomic ratio	Ce:Ir = 1.003		Ce:Ir = 0.737	

References

1. Gao, J.; Xu, C.-Q.; Hung, S.-F.; Liu, W.; Cai, W.; Zeng, Z.; Jia, C.; Chen, H. M.; Xiao, H.; Li, J.; Huang, Y.; Liu, B., Breaking long-range order in iridium oxide by alkali ion for efficient water oxidation. *J. Am. Chem. Soc.* **2019,** 141, 3014-3023.
2. Li, G.; Li, K.; Yang, L.; Chang, J.; Ma, R.; Wu, Z.; Ge, J.; Liu, C.; Xing, W., Boosted performance of Ir species by employing TiN as the support toward oxygen evolution reaction. *ACS Appl. Mater. Interfaces* **2018,** 10, 38117-38124.
3. Wu, X.; Feng, B.; Li, W.; Niu, Y.; Yu, Y.; Lu, S.; Zhong, C.; Liu, P.; Tian, Z.; Chen, L.; Hu, W.; Li, C. M., Metal-support interaction boosted electrocatalysis of ultrasmall iridium nanoparticles supported on nitrogen doped graphene for highly efficient water electrolysis in acidic and alkaline media. *Nano Energy* **2019,** 62, 117-126.
4. Zhang, H.; Shi, P.; Ma, X.; Ma, C.; Han, S.; He, C.; Wu, H.; Zhu, L.; Zhang, B.; Lu, Y.; Cao, W.; Yin, H.; Meng, X.; Xia, J.; Zhang, J.; Wang, A.-L.; Lu, Q., Construction of ordered atomic donor-acceptor architectures in bcc IrGa intermetallic compounds toward highly electroactive and stable overall water splitting. *Adv. Energy Mater.* **2022,** 13, 2202703.
5. Zhang, L.; Jang, H.; Li, Z.; Liu, H.; Kim, M. G.; Liu, X.; Cho, J., $SrIrO_3$ modified with laminar Sr_2IrO_4 as a robust bifunctional electrocatalyst for overall water splitting in acidic media. *Chem. Eng. J.* **2021,** 419, 129604.
6. Zheng, X.; Qin, M.; Ma, S.; Chen, Y.; Ning, H.; Yang, R.; Mao, S.; Wang, Y., Strong oxide-support interaction over IrO_2/V_2O_5 for efficient pH-universal water splitting. *Adv. Sci.* **2022,** 9, 2104636.
7. Zhang, Y.; Zhang, G.; Zhang, M.; Zhu, X.; Shi, P.; Wang, S.; Wang, A.-L., Synergistic electronic and morphological modulation by trace Ir introduction boosting oxygen evolution performance over a wide pH range. *Chem. Eng. J.* **2022,** 433, 133577.
8. Shan, J.; Guo, C.; Zhu, Y.; Chen, S.; Song, L.; Jaroniec, M.; Zheng, Y.; Qiao, S.-Z., Charge-redistribution-enhanced nanocrystalline Ru@IrO_x electrocatalysts for oxygen evolution in acidic media. *Chem* **2019,** 5, 445-459.
9. Chen, X.; Liao, W.; Zhong, M.; Chen, J.; Yan, S.; Li, W.; Wang, C.; Chen, W.; Lu, X., Rational

design of robust iridium-ceria oxide-carbon nanofibers to boost oxygen evolution reaction in both alkaline and acidic media. *Nano Res.* **2022,** 16, 7724-7732.

10. Seitz, L. C.; Dickens, C. F.; Nishio, K.; Hikita, Y.; Montoya, J.; Doyle, A.; Kirk, C.; Vojvodic, A.; Hwang, H. Y.; Norskov, J. K.; Jaramillo, T. F., A highly active and stable $IrO_x/SrIrO_3$ catalyst for the oxygen evolution reaction. *Science* **2016,** 353, 1011-1014.
11. Chen, X.; Li, W.; Song, N.; Zhong, M.; Yan, S.; Xu, J.; Zhu, W.; Wang, C.; Lu, X., Electronic modulation of iridium-molybdenum oxides with a low crystallinity for high-efficiency acidic oxygen evolution reaction. *Chem. Eng. J.* **2022,** 440, 135851.
12. Nong, H. N.; Reier, T.; Oh, H.-S.; Gliech, M.; Paciok, P.; Vu, T. H. T.; Teschner, D.; Heggen, M.; Petkov, V.; Schlögl, R.; Jones, T.; Strasser, P., A unique oxygen ligand environment facilitates water oxidation in hole-doped $IrNiO_x$ core–shell electrocatalysts. *Nat. Catal.* **2018,** 1, 841-851.
13. Zhu, M.; Shao, Q.; Qian, Y.; Huang, X., Superior overall water splitting electrocatalysis in acidic conditions enabled by bimetallic Ir-Ag nanotubes. *Nano Energy* **2019,** 56, 330-337.
14. Grimaud, A.; Demortiere, A.; Saubanere, M.; Dachraoui, W.; Duchamp, M.; Doublet, M.-L.; Tarascon, J.-M., Activation of surface oxygen sites on an iridium-based model catalyst for the oxygen evolution reaction. *Nat. Energy* **2016,** 2, 16189.
